# Parenting Sense of Competence Scale (PSOC): Establishing Normative Scores in Mothers of Infants Under 9 Months

**DOI:** 10.3390/children13040523

**Published:** 2026-04-09

**Authors:** Gemma Pons-Salvador, Rosa M. Trenado, Lucía Ballabriga-Olivito

**Affiliations:** 1 Aggression and Family Research Group, Department of Psychology, University of Valencia, 46022 Valencia, Spain; rosa.m.trenado@uv.es; 2 Aggression and Family Research Group, University of Valencia, 46022 Valencia, Spain; lubao@alumni.uv.es

**Keywords:** Parenting Sense of Competence scale (PSOC), normative scores, percentiles, Z- and T-scores, mothers of infants under 9 months old

## Abstract

**Highlights:**

**What are the main findings?**

- PSOC norms are provided for Spanish mothers with infants under 9 months for early assessment.
- Parental competence drops after 9 months, and mothers over 35 show lower self-efficacy.

**What are the implications of the main findings?**

- These norms help identify risk profiles and inform the development of tailored early-intervention strategies.
- They highlight the PSOC’s value for early screening and monitoring, supporting its use in clinical, psychoeducational, and research contexts.

**Abstract:**

Background/Objectives: The Parenting Sense of Competence (PSOC) scale is one of the most widely used instruments to assess perceived parental competence, understood as the degree to which parents feel capable of adequately fulfilling their parental role. Despite its widespread use, studies seeking to determine PSOC normative scores are scarce, especially in specific populations such as mothers with infants younger than 9 months, which limits the interpretation of its scores in applied contexts. This study establishes PSOC normative scores in a nonclinical sample of 522 Spanish mothers with infants aged between 3 and 37 weeks who attended a public early intervention program. Methods: Regression and ANOVA analyses were performed to examine the effect of infant and maternal age, as well as educational level and occupation, on the dimensions of Efficacy, Satisfaction, and Total score of the PSOC. Results: The results show a significant decline in parental competence starting when their infants reach 9 months of age, and lower levels of self-efficacy in mothers over 35 years of age. No significant differences were found according to the educational level or occupation of the mothers. Normative scores are presented by percentiles, offering specific criteria for this stage of child development. Z- and T-scores are included, useful for standardized comparisons between subscale and studies. Conclusions: These findings provide useful information for early detection and psychoeducational interventions within the framework of early intervention.

## 1. Introduction

The perception of parental competence refers to the degree to which parents feel effective and satisfied in their parental role, and has been linked to multiple indicators of family well-being and child development [1]. It is a key construct for understanding the psychological adjustment of parents and its influence on the quality of the family environment [2]. Its relevance is especially significant during the first year of a child’s life, as it influences parenting practices, emotional bonding, and children’s emotional development [3]. Research in the perinatal population shows that greater parental self-efficacy predicts better parenting behaviors and outcomes in babies’ development [4]. This perception takes on special relevance in contexts of psychosocial risk, where parenting is especially difficult, given that parental competence acts as a protective factor against environmental adversities [5].

In recent years, early parenting has increasingly been conceptualised as a dynamic, dyadic, and biologically grounded process. Within this framework, the emotional experiences of the parent and infant are organised through interaction and mutual co-regulation [6,7]. From this perspective, perceived parental competence should be understood not only as an individual belief about one’s own ability, but also as a subjective experience constructed through repeated interactions with the child. These interactions include parental sensitivity, the ability to soothe and interpret signals, and the ability to adjust to the child’s needs [8]. In line with this view, recent work in developmental neuroscience has emphasised that attentional, neural, and physiological synchrony within the parent–infant dyad constitutes an important basis for emotional regulation and the organisation of early affective experience [7].

To assess this perception of parental competence, one of the most widely used instruments is the Parenting Sense of Competence (PSOC) scale, originally developed by Gibaud-Wallston and Wandersman (1978, cited in Johnston and Mash, 1989) and later published and validated by Johnston and Mash [9]. The PSOC makes it possible to assess the subjective perception of parental efficacy and satisfaction, which, although measured through self-report, may be understood as partially linked to everyday experiences of interaction and co-regulation within the dyad. This questionnaire enables the assessment of two central dimensions of the construct: Efficacy (feeling of control and ability to cope with the demands of parenting) and Satisfaction (degree of enjoyment and contentment with the parental role). Its versatility and conciseness have favored its use across research, clinical, and intervention environments [1].

Indeed, in recent decades, the PSOC has been widely used in studies with parents of preschool or school-aged children [10], as well as in specific contexts, such as first-time mothers [3], parents of premature children [11,12], or children with developmental disorders, such as ADHD or ASD [13,14]. It has also been used in cross-cultural research [15]. Together, these studies show the versatility of the PSOC as a tool to assess parental self-efficacy in a wide variety of family and cultural contexts, as well as its usefulness in designing interventions tailored to the specific needs of parents.

At the same time, an increasing number of studies have focused on factors that influence the perception of parental efficacy, such as maternal depression [16,17], anxiety [18], maternal self-esteem [19,20], the availability of social support [16,21], and the number of children [22], among others. These studies show that parental self-efficacy is not only shaped by the demands of childcare, but is also influenced by personal, emotional, and contextual factors.

In addition to these individual and relational variables, the role of sociodemographic factors in the perception of parental competence has also been investigated, although the findings are less consistent. Regarding maternal age, there is no clear consensus on its effect: some studies find a positive, albeit weak, correlation [16,23], while others observe a negative relationship [24,25]. These differences could be due to associated factors, such as social support. For example, young mothers with support networks, especially from their own mothers, showed higher levels of parental competence [20,26]. In the case of older mothers with partners, marital satisfaction, as an indirect indicator of social support, was positively associated with parental self-efficacy [21,27].

There is also disagreement about the role of socioeconomic status. A recent study of parents experiencing adversity found that self-reported financial perceptions were significantly related to parental self-efficacy, while educational level did not show a clear influence [28]. This finding suggests that financial hardship may undermine parental competence by exacerbating stress or social exclusion. Cerezo et al. [27] also found that mothers without paid employment perceived lower competence, particularly regarding satisfaction with the parental role. However, other studies have not found significant associations, possibly due to methodological, sampling, or cultural context variations [25].

In addition to the characteristics of mothers and their context, children’s age has also been observed to influence the perception of parental competence. Although the PSOC has traditionally been applied in large samples of parents with children of different ages, some studies have revealed significant differences depending on the children’s age. Gilmore & Cuskelly [29] observed that mothers of infants (0–3 years) and adolescents (13–17 years) showed greater self-efficacy than those with children aged between 8 and 12 years. Furthermore, the latter scored lower on the parental satisfaction subscale than the rest of the groups. These results underline the importance of considering the developmental stage of childhood when assessing parental competence. In this regard, some studies have focused on specific stages such as early childhood, due to its particularly high demands in terms of parental adaptation. For example, Karp et al. [16] analyzed factors associated with parenting during the first year of life in mothers with children aged 2 to 12 months. In Spain, Oltra-Benavent et al. [25] confirmed the validity and reliability of the adapted PSOC, translating the instrument into Spanish, in a sample of mothers with babies aged 6 to 12 months.

From an applied perspective, the PSOC has been used as an assessment tool in research on the effectiveness of programs aimed at parents with infants, focused on promoting child well-being and preventing parenting difficulties. Some of these studies have shown significant changes in parental competence and perceived self-efficacy after participation in these interventions [27,30,31]. Based on these findings, these authors have highlighted the importance of developing specific assessment studies for specific populations, which facilitate more accurate clinical assessments within the framework of preventive and parenting support programs. Although assessment studies are rare because they require large and representative samples, these analyses are essential to provide objective criteria to assess whether a score is high, low, or average relative to a reference population, avoiding arbitrary interpretations. This information is essential to define clear assessment guidelines, guide evidence-based clinical decisions, and identify atypical profiles early.

### The Current Study

Our research focuses on establishing normative data for the Parenting Sense of Competence (PSOC) scale in a large sample of mothers of infants under the age of 9 months. From 9 months onward, significant changes occur in child development, implying new challenges in how parents experience their parental role, which can affect their perception of their own competence.

The study also compares mothers according to certain sociodemographic variables, such as age, educational level, and occupation. The proposed hypotheses are exploratory in nature, given that, as noted, the literature does not offer a clear consensus on these variables. The main objective of these comparative analyses is to describe the characteristics of the evaluated maternal cohort with greater precision.

## 2. Materials and Methods

### 2.1. Participants

The normative study included a sample of 522 mothers of babies between 3 and 37 weeks old (babies under the age of 9 months). The ages of the mothers ranged from 18 to 44 years (*M* = 33.75, *SD* = 4.10). Most had a medium to high level of education: 43% of mothers (*n* = 244) had higher education (university), 31% (*n* = 176) had an intermediate level, which included vocational training or high school, while 12.9% (*n* = 73) had basic training in primary or compulsory secondary education, or were still students. In 13% of cases (*n* = 74), they had not completed basic education. Regarding maternal occupation, 34.48% (*n* = 180) were classified as scientific or intellectual professionals or in skilled management and leadership positions, and 38.31% (*n* = 200) as technical or support professionals, administrative, industrial, or service-related. The remaining occupations were less frequent or unclassifiable. A high percentage of mothers (24.52%, *n* = 128) were unemployed at the time of data collection, which in some cases could be linked to postnatal termination. Cases with no information (*n* = 14) were considered missing data and excluded from the analyses. No imputation techniques were applied due to the low percentage (2.7%, *n* = 14) of missing data. Although this low percentage suggests a limited impact on the results, a potential bias associated with this decision cannot be completely ruled out.

### 2.2. Procedures

The information analyzed in this study was extracted from the initial interview conducted with parents who attended a public early intervention program aimed at families with infants under two years of age. The program is aimed at the entire population, and parents attend voluntarily. During this interview, the Parenting Sense of Competence (PSOC) [9] questionnaire was administered, among other instruments. In addition, relevant sociodemographic data was collected. In most cases, the mother attended the interview, so the present assessment study focused solely on the maternal population.

In the initial data collection, 568 completed PSOC questionnaires were obtained from mothers with infants aged between 3 and 101 weeks (*M* = 22.58; *SD* = 13.26). The primary objective of the study was to assess the Spanish version of the PSOC in a sample of mothers with infants under 2 years of age. As a first step, the homogeneity of the sample in relation to the age of the infants (in weeks) was analyzed. The results presented in the preliminary analyses section showed significant differences in PSOC scores between mothers of infants younger than 9 months and those with infants older than 9 months. Based on these findings, the assessment study was conducted exclusively with a subsample of 522 mothers whose infants were younger than 9 months (between 3 and 37 weeks of age). Additionally, an analysis of PSOC scores was performed with this sample, considering various sociodemographic characteristics of the mothers. The normative study was not conducted with mothers of infants older than 9 months because the number in this group was insufficient for this type of analysis.

The study was conducted in accordance with professional ethical guidelines, including obtaining informed consent from participants, ensuring ethical treatment and respect for their rights, and safeguarding their privacy and personal data so that individual participants could not be identified either in the reported findings or through publicly available original or archived data.

### 2.3. Instruments

The Parenting Sense of Competence (PSOC) scale, developed by Gibaud-Wallston and Wandersman (1978; cited in Johnston & Mash, 1989) [9], is a self-report tool that measures perceived parental competence. Johnston and Mash later adapted and published the instrument in 1989 [9]. The PSOC uses a 6-point Likert scale (from 1 = strongly disagree to 6 = strongly agree), and the total score is calculated by adding two subscales: Satisfaction (9 items related to anxiety, frustration, and motivation in parenting) and Perceived Self-Efficacy (8 items related to skills and problem-solving). In its original version, the instrument demonstrated adequate reliability (*α* = 0.70 for efficacy and *α* = 0.82 for satisfaction).

The PSOC has been widely used and is one of the most validated in different contexts and countries [22,23,32], being one of the most used to measure perceived parental competence [1,33], thanks to its brevity and psychometric robustness. Several studies have adapted and validated this scale in Spanish, with modifications such as the reduction in items [34] with adequate internal consistency [35] or improvements in the wording to facilitate understanding [25]. This latest version, carried out with mothers of children aged 6 to 12 months, has shown high internal consistency (*α* = 0.85) and convergent validity, with positive associations with dyadic adjustment and negative associations with depression, fatigue, and parental stress. Recent evidence maintains adequate consistency for the total scale with foster parents of children between 4 and 9 years old (*α* = 0.83) [32].

In the present study, the Spanish translation of the original version is used, using the scores of both subscales and the total as continuous variables, obtaining good internal consistency with *α* = 0.82.

### 2.4. Variables

The Parenting Sense of Competence (PSOC) scale and its subscales were included as dependent variables. The independent variables considered were the age of the infants, the age of the mothers, the educational level, and the occupation of the mother.

To conduct a preliminary comparative study between groups of mothers according to the age of the babies, they were classified as: (1) 0 to 3 months, (2) 3 to 6 months, (3) 6 to 9 months, (4) over 9 months.

Regarding the age of the mothers, the classification was: under 35 years (18 to 34 years) and over 35 years (35 to 44 years). This classification was based on the median, which was 34 years.

In terms of the mothers’ highest educational level, they were classified for group comparisons as follows: High Level: higher education, university, master’s degree, doctorate; Medium Level: high school, vocational training; and Low Level: primary education, compulsory secondary education (ESO), EGB (Bachelor’s degree), or “studying” status.

The mothers’ occupation at the time of the assessment was classified according to the major groups of the International Standard Classification of Occupations (ISCO-08), using the adapted Spanish version [36]. This classification groups occupations into ten large categories coded by one digit (large groups), allowing for standardized interpretation and international comparability. Based on this classification, mothers were grouped into three levels: High Level (High Income): professions that typically require university or higher technical training, with high stability or high salaries; Medium Level (Medium Income): skilled occupations with less stability or average salaries; Low Level (Low Income or Vulnerable Situation): occupations with low stability, low economic remuneration, or unemployment. The Low Level includes mothers who are not actively employed, i.e., those who were not working outside the home at the time of the assessment.

### 2.5. Data Analysis

Descriptive statistics (means, standard deviations, minimums, and maximums) were calculated for the total score and the two PSOC subscales. Based on the score distribution, percentile scales were developed (5th percentile–95th percentile, every 5 points), including transformations to Z- and T-scores.

Linear regression analyses of continuous variables, such as infant and maternal age, were conducted. For comparisons between groups on the different variables, an ANOVA was conducted to compare the three infant age groups, and *t*-tests were performed to compare the two maternal age groups. A MANOVA was also conducted to study the effect of the mother’s educational level and occupation, as well as their interaction, on the three dimensions of parental competence.

The alpha value was 0.05. The assumption of homogeneity was tested by calculating the Levene variance test. Bonferroni *t* tests were used for post hoc analysis.

The Eta squared (*η*^2^) was interpreted according to the reference values proposed by Cohen [37]: 0.01 (small), 0.06 (medium), and 0.14 (large). All analyses were performed using SPSS v.20.0 for Windows.

## 3. Results

### 3.1. Preliminary Analysis, According to the Age of the Infant

Simple Linear Regression analyses were conducted to examine the effect of infant age, measured in weeks, on the three dimensions of perceived parental competence: Efficacy, Satisfaction, and Total (sum of Efficacy and Satisfaction). This preliminary analysis was performed on the initial sample of *N* = 568. In this case, infants ranged in age from 3 weeks to 101 weeks (*M* = 22.58, *SD* = 3.26). Detailed results for each subscale are presented in Table 1. The findings indicate a significant decline in parental competence (both Total and the Efficacy and Satisfaction subscales) with each passing week of the infant’s development, although the explained variance *R*^2^ was low in all three cases.

Considering the previous results of the linear regressions and the literature indicating that a baby’s autonomous mobility can affect parental self-competence perception, an ANOVA was performed to compare the PSOC scores (Total, Efficacy, and Satisfaction) among subjects divided into age groups: (1) 0 to 3 months; (2) 3 to 6 months; (3) 6 to 9 months; and (4) more than 9 months.

The results showed that in both the Efficacy and Satisfaction subscales, as well as the Total scale of the PSOC, the lowest averages were found for mothers with babies older than 9 months, with significant differences in PSOC-Efficacy and PSOC-Total (Table 2). Although the effect sizes were small, post hoc analyses confirmed that significant differences occurred in PSOC-Efficacy between the 9+ month group and the 3–6 month group (*t* = 3.17, *p* = 0.004, Bonferroni = 0.005) and the 6–9 month group (*t* = 2.91, *p* = 0.027, Bonferroni = 0.032). There were also significant differences in PSOC-Total between the 9+ month group and the 0–3 month group (*t* = 7.90, *p* = 0.019, Bonferroni = 0.021), the 3–6 month group (*t* = 10.8, *p* < 0.001, Bonferroni < 0.001), and the 6–9 month group (*t* = 8.14, *p* = 0.006, Bonferroni = 0.006).

Based on these findings, we determined that only infants between 3 and 37 weeks of age, or 0 to 9 months, would be included in the current study. Applying this selection criterion, the sample for the study of normative scores totaled *N* = 522.

### 3.2. Sociodemographic Characteristics of Mothers

We considered whether there were significant differences in PSOC-Efficacy, Satisfaction, and Total among mothers based on certain sociodemographic characteristics such as age, educational level, and occupation in the final study sample of 522 mothers.

#### 3.2.1. Mother’s Age

Three simple linear regression analyses were conducted to examine whether the mother’s exact age significantly predicted scores on the Efficacy, Satisfaction, and Total subscales (Table 3). The results suggest that maternal age has a significant, negative relationship with overall perceptions of parental competence, particularly in the Efficacy dimension. However, the relationship is weak, and the effect size is small. No significant associations were observed with the Satisfaction subscale.

Considering the previous results, mothers were distributed into two groups: mothers under 35 years of age (18 to 34 years old, *M* = 30.89, *SD* = 2.99, *n* = 285) and mothers over 35 years of age (35 to 44 years old, *M* = 37.23, *SD* = 2.09, *n* = 234). The results of the comparisons of means showed that there were significant differences between these two groups in Efficacy (*t* = 3.58, *p* < 0.001) and Total (*t* = 2.75, *p* = 0.003), but not in Satisfaction (*t* = 0.84, *p* = 0.20) (Table 4). Younger mothers perceived greater efficacy and greater total parental competence than older mothers. Although the differences are statistically significant in the Efficacy dimension and the Total scale, the effect size is small to moderate, indicating that the differences, while real, are not of great magnitude in practical terms.

#### 3.2.2. Mother’s Educational Level and Occupation

A MANOVA was used to study the effect of the mother’s educational level and occupation, as well as their interaction, on the dimensions of parental competence: Efficacy, Satisfaction and Total. The results, collected in Table 5, showed that there are no significant overall differences in perceived parental competence (Efficacy, Satisfaction and Total) depending on the mother’s educational level or occupation. Only the interaction between educational level and occupation significantly affects the perception of parental efficacy, but not the Satisfaction dimension or Total scale. The differences detected are small and the effect size is also small (*η*^2^ = 0.028), so these differences should be interpreted with caution. In fact, no significant differences were shown in the post hoc analyses.

### 3.3. Analysis of the Normative Scores of PSOC Total and Each of Its Subscales

#### 3.3.1. Descriptive Analysis

The analysis of normative scores was performed for the entire sample (*N* = 522 subjects), that is, for mothers with infants aged 0 to 9 months. Table 6 shows the means, standard deviations, medians, and minimum and maximum scores achieved.

#### 3.3.2. Analysis of Percentiles

The results from the assessment of the 5th to 95th percentiles concerning the dimensions of PSOC-Efficacy, PSOC-Satisfaction, and PSOC-Total are shown in Table 7.

In order to facilitate the practical use of this Table 7, the interpretation of the norm-referenced percentiles by subscale is shown below:

- PSOC-Efficacy Percentiles

This subscale measures the perception of efficacy in the parental role, that is, how competent the person feels when facing parenting tasks.

✓ Low scores (below the 25th percentile, i.e., ≤32) may indicate a reduced perception of control, security, or competence as a parent.
✓ Average scores (25–75th percentiles, between 33 and 40) reflect a moderate or adequate perception of parental competence.
✓ High scores (above the 75th percentile, ≥41) indicate a strong perception of self-efficacy and confidence in role performance.

- PSOC-Satisfaction Percentiles

This subscale evaluates the level of emotional satisfaction and subjective well-being associated with the parental role.

✓ Low scores (≤38) may be associated with stress, frustration, or discouragement with parenting.
✓ Medium scores (between 39 and 46) indicate a moderate level of satisfaction with the role.
✓ High scores (≥47) reflect high satisfaction and enjoyment of the parental role.

- PSOC-Total Percentiles

This dimension is an integrated measure that combines parental efficacy and satisfaction.

✓ Low scores (≤72) may suggest general difficulties in adjusting to the parental role.
✓ Average scores (between 73 and 84) are considered within the expected range for most parents.
✓ High scores (≥85) indicate a strong sense of parental competence and well-being.

#### 3.3.3. Analysis of the Normative Scores

To allow for consistent comparisons among subscales and research investigations, this section presents the findings for the Z- and T-scores, which are categorized by the percentiles analyzed from 5 to 95 (refer to Table 8, Table 9 and Table 10).

Z-scores directly indicate a result’s distance from the mean in standard deviations, thus offering a precise, but sometimes less intuitive, reference for practical interpretation. For this reason, it is useful to complement them with T-scores, which are normalized to a mean of 50 and a standard deviation of 10, enabling a simpler and more uniform interpretation. Values around *T* = 50 indicate an average level of efficacy, satisfaction, or total score, whereas values below *T* = 40 reflect low levels (equivalent to one standard deviation below the mean), and values equal to or greater than *T* = 60 indicate high levels. In this way, T-scores not only expand the information provided by percentiles but also establish a stable and replicable comparison framework in research studies and professional application contexts.

Considering the total PSOC scores, values equal to or less than 63 correspond to the 5th percentile (*T* = 34.7), whereas scores equal to or greater than 91 correspond to the 95th percentile (*T* = 64.3). Thus, low scores reflect a clearly reduced perception of parental competence, while high scores indicate a very high level of perceived competence.

## 4. Discussion

This study examines how mothers of infants under 9 months perceive their parental competence, utilizing the Parenting Sense of Competence (PSOC) scale within a Spanish sample. The main objective was to establish normative standards tailored to this age group, using a large and representative sample, given that existing studies on PSOC have not previously offered specific criteria for this period of child development. Furthermore, potential differences based on sociodemographic variables, such as maternal age, educational level, and occupation were explored, to better characterize the participating mothers and provide empirical data on factors associated with parental competence at this early stage.

In a previous analysis, carried out with mothers of infants up to 24 months, we observed that parental competence, especially the Efficacy subscale, decreases significantly from 9 months onward. This decline coincides with the infant’s increasing motor and communicative autonomy and with the emergence of separation anxiety, suggesting that this pattern may be interpreted as a process of dyadic reorganisation. Developmental advances characteristic of this stage increase the regulatory demands placed on the mother, who must adapt to new needs related to emotional support, behavioural regulation, and interactive coordination. This interpretation is consistent with studies showing that new developmental challenges in infancy increase parental demands and may affect perceptions of self-efficacy [4,29]. At the same time, early patterns of co-regulation that may have supported maternal perceptions of efficacy during the first months may no longer be sufficient, requiring the dyad to develop new forms of mutual adjustment. In this regard, and in line with Leerkes and Crockenberg [3] and Karp et al. [16], our results reinforce the idea that perceptions of parental competence in the early stages are particularly sensitive both to the infant’s developmental changes and to the reorganisation of mother–child interactive processes. From this perspective, the observed decline in perceived parental competence would reflect not only a loss of confidence, but also the adaptive challenge associated with the transition to a new phase of infant development.

Regarding maternal age, our data show that younger mothers report significantly higher levels of total parental competence and efficacy, although the differences found are small to moderate. This negative relationship between maternal age and perceived competence partially coincides with some studies that identified similar associations [24,25] and could be explained by related variables, such as the higher level of social support available to some young mothers [20,26]. However, the absence of significant differences in the Satisfaction subscale suggests that maternal age could be mainly linked to the self-efficacy component rather than the affective component of the parental role.

As for sociodemographic variables, such as educational level and occupation, the results revealed no relevant differences in the perception of parental competence. Although a statistically significant interaction was observed between both variables for perceived efficacy, this result appears to have limited practical relevance given its small effect size. Taken together, the absence of relevant differences across educational level and occupation, suggests that parental competence in early childhood may be more closely linked to proximal relational, emotional, and contextual processes than to structural indicators such as education or occupation considered in isolation. From a developmental perspective, this interpretation is consistent with the idea that the everyday demands of caregiving, the quality of interactions, and the subjective experience of support and competence are particularly relevant to the development of parental self-efficacy in the early years of life. Studies such as that of Kaasbøll et al. [28] indicate that the subjective perception of economic adversity may have a greater impact on parental competence than objective structural indicators, such as educational level. Similarly, our results suggest that proximal factors such as social support or maternal emotional well-being may play a more decisive role in perceptions of parental self-efficacy and satisfaction than sociodemographic variables per se [21,27]. Taken together, these findings support the hypothesis that structural conditions may exert an indirect influence in early developmental stages, whereas more immediate relational and emotional processes may play a more direct role in shaping parental competence.

However, given that the primary objective of the study was the assessment of the PSOC, the contribution of normative values obtained is especially relevant. These allow for a more accurate assessment of parental competence in mothers with infants aged 0 to 9 months and respond to the need identified in previous research [27,30,31]. Interpreting PSOC scores based on percentiles provides clear clinical utility in early intervention. It allows risk profiles to be identified more precisely and helps to tailor family support strategies. In particular, scores below the 25th percentile may indicate potential difficulties in parental competence, suggesting the need for a more detailed assessment and specific parenting support interventions to promote child and family well-being.

We also included analyses of Z- and T-scores in the study. For clarity and applicability, we recommend using percentiles in clinical or preventive settings, where communication with professionals and families should be accessible. For comparative or research studies, T-scores offer methodological advantages by allowing standardized comparisons across subscales and studies.

Taken together, the findings of this study underscore the importance of considering both the infant’s characteristics (age and developmental stage) and the mother’s sociodemographic and contextual profile when assessing perceived parental competence. Furthermore, they reinforce the usefulness of the PSOC as a screening and monitoring instrument at sensitive stages of development, supporting its use in clinical, psychoeducational, and research settings, as multiple studies have pointed out [1,11,27,38,39].

### Limitations and Future Lines

This work also presents some limitations that should be noted, and that may guide future research. On the one hand, Although the sample size was large, participation in the public early intervention programme was voluntary, which may have introduced a self-selection bias. Therefore, the sample cannot be considered strictly representative of the general population. On the other hand, although some sociodemographic variables were analyzed, other relevant dimensions such as perceived social support or maternal mental health status were not included, which the literature has identified as key factors in parental competence [16,18,28]. Along these lines, future studies could expand the analysis of parental competence through longitudinal designs that allow observing its evolution throughout the first year of life, as well as exploring the role of contextual and psychosocial factors that modulate this perception, such as the bond with the baby, the mother’s emotional health, or the presence of support networks.

A major limitation of the study is that it was conducted solely with mothers, given that insufficient information was available from fathers. This implies that, as examined here, parental competence is defined on the basis of maternal experience. Therefore, it cannot be generalised to parental competence in a broader sense without caution. Future research would be of great interest if it also included adequate data from male parents. It would also be important to develop specific scales for other age groups of children, to improve the diagnostic sensitivity of the instrument and its applicability in various family prevention and intervention programs.

## 5. Implications for Practice and Theory

This study provides, for the first time in Spain, specific PSOC normative scores for mothers with infants up to 9 months old, developed from a large and contextualized sample. Their availability constitutes a valuable resource for clinical assessment and research in early intervention, facilitating the detection of risk profiles and the planning of tailored support.

In practical terms, these findings highlight the importance of considering both infant and maternal characteristics when examining the perception of parental competence, and of integrating standardized instruments such as the PSOC into early screening protocols. This reinforces the value of preventive interventions aimed at promoting child and family well-being from the earliest stages of development.

At the same time, our findings suggest that the assessment of parental competence may benefit from a broader approach, in which the self-perception measured by the PSOC is complemented by dyadic and interactional indicators that contextualise this perception within the mother–infant relationship. From this perspective, early intervention should not focus solely on strengthening parental self-efficacy and satisfaction, but also on addressing the relational and mutual adjustment processes that shape the experience of parenting in the early years. Thus, the PSOC may be a valuable tool not only for identifying risk profiles, but also for guiding more integrative assessment and intervention strategies.

## Figures and Tables

**Table 1:** Linear regressions between infant age and PSOC scores (*N* = 568).

PSOC Subscale	*R*	*R* ^2^	*F*	*p*	*B* (Age)	*p*
Efficacy	0.10	0.01	5.90 *	0.015	−0.046 *	0.015
Satisfaction	0.10	0.01	6.65 *	0.010	−0.049 *	0.010
Total	0.14	0.02	12.52 *	<0.001	−0.160 *	<0.001

* Significant correlation *p* < 0.005.

**Table 2:** ANOVA results for PSOC scores by infant age (*N* = 568).

PSOC Subscales	*N*	*M*	*SD*	*F*	*p*	Partial *η*^2^
Efficacy				3.93 *	0.009	0.020
0–3 months	68	35.43	6.47			
3–6 months	341	36.21	5.92			
6–9 months	114	35.96	5.25			
>9 months	45	33.04	6.53			
Satisfaction				2.26	0.081	0.012
0–3 months	68	42.07	5.94			
3–6 months	341	41.37	6.03			
6–9 months	114	41.12	5.81			
>9 months	45	39.18	6.65			
Total				4.31 *	0.005	0.022
0–3 months	71	74.23	18.57			
3–6 months	343	77.13	11.22			
6–9 months	118	74.47	16.39			
>9 months	49	66.33	22.04			

* Significant correlation *p* < 0.005.

**Table 3:** Linear regressions between maternal age and PSOC scores (*N* = 522).

Dependent Variable	*R*	*R* ^2^	*F*	*p*	*B* (Age)	*p*
PSOC Efficacy	0.159	0.025	13.46 *	<0.001	−0.224 *	<0.001
PSOC Satisfaction	0.022	0.0	0.26	0.613	−0.032	0.613
PSOC Total	0.113	0.013	6.69 *	0.010	−0.255 *	0.010

* Significant correlation *p* < 0.005.

**Table 4:** Comparison of PSOC mean scores by maternal age group (*N* = 522).

PSOC Subscales	Group 1 (15–34 Years)	Group 2 (35–44 Years)	Average Difference	*t*	*p*	Cohen’s *d*
Efficacy	36.88	35.05	1.83	3.58 *	<0.001	0.316
Satisfaction	41.63	41.19	0.44	0.84	0.200	0.074
Total	78.51	76.24	2.28	2.75 *	0.003	0.243

* Significant correlation *p* < 0.005.

**Table 5:** MANOVA results for PSOC scores by maternal educational level and occupation (*N* = 522).

PSOC Subscale		*M*	*SD*	*F*	*p*	*η* ^2^
Efficacy	Educational level			0.31	0.735	0.001
	High	35.39	5.65			
	Middle	36.63	5.80			
	Low	37.18	6.01			
Satisfaction	Educational level			0.01	0.986	0.000
	High	41.74	5.59			
	Middle	41.84	6.21			
	Low	40.55	5.62			
Total	Educational level			0.02	0.891	0.001
	High	77.13	8.68			
	Middle	78.47	9.83			
	Low	77.74	9.57			
		*M*	*SD*	*F*	*p*	*η* ^2^
Efficacy	Occupation			0.32	0.726	0.001
	High	35.47	6.47			
	Middle	36.13	5.53			
	Low	36.32	6.02			
Satisfaction	Occupation			0.44	0.645	0.002
	High	42.07	6.14			
	Middle	41.46	5.87			
	Low	40.87	6.02			
Total	Occupation			0.06	0.937	0.000
	High	77.53	9.47			
	Middle	77.62	9.29			
	Low	77.19	9.84			
		*M*	*SD*	*F*	*p*	*η* ^2^
Efficacy	Education × Occupation			3.16 *	0.014	0.028
	High	35.39	5.65			
	Middle	36.63	5.81			
	Low	37.08	5.99			
Satisfaction	Education × Occupation			0.04	0.789	0.004
	High	41.74	5.60			
	Middle	41.84	6.21			
	Low	40.69	5.62			
Total	Education × Occupation			2.057	0.085	0.018
	High	77.13	8.68			
	Middle	78.47	9.83			
	Low	77.77	9.64			

* Significant correlation *p* < 0.005.

**Table 6:** Descriptive statistics for PSOC scores (*N* = 522).

Subscale	Average	*SD*	Median	Minimum	Maximum
PSOC Efficacy	36.06	5.86	36.5	13	53
PSOC Satisfaction	41.41	5.97	42	9	55
PSOC Total	77.47	9.44	78	22	101

**Table 7:** Percentile norms on the PSOC (Efficacy and Satisfaction subscales and Total score) in mothers with infants aged 0 to 9 months.

Percentile	PSOC Efficacy	PSOC Satisfaction	PSOC Total
5	26.0	32.0	63.0
10	28.0	34.0	66.1
15	30.0	36.0	68.0
20	31.0	37.0	70.0
25	32.0	38.0	72.0
30	33.0	38.0	73.0
35	34.0	40.0	74.0
40	35.0	40.0	76.0
45	36.0	41.0	77.0
50	36.5	42.0	78.0
55	37.0	43.0	79.0
60	38.0	43.0	80.0
65	38.0	44.0	81.0
70	39.0	45.0	82.0
75	40.0	46.0	84.0
80	41.0	46.0	85.0
85	42.0	47.0	87.0
90	44.0	49.0	89.0
95	45.0	50.0	91.0

**Table 8:** Normative data for the Total PSOC Score in mothers with infants aged 0–9 months (percentiles, raw, Z, and T scores).

Percentile	Raw Core	*Z*	*T*
5.0	63.0	−1.53	34.7
10.0	66.1	−1.21	37.9
15.0	68.0	−1.0	40.0
20.0	70.0	−0.79	42.1
25.0	72.0	−0.58	44.2
30.0	73.0	−0.47	45.3
35.0	74.0	−0.37	46.3
40.0	76.0	−0.16	48.4
45.0	77.0	−0.05	49.5
50.0	78.0	0.06	50.6
55.0	79.0	0.16	51.6
60.0	80.0	0.27	52.7
65.0	81.0	0.37	53.7
70.0	82.0	0.48	54.8
75.0	84.0	0.69	56.9
80.0	85.0	0.8	58.0
85.0	87.0	1.01	60.1
90.0	89.0	1.22	62.2
95.0	91.0	1.43	64.3

**Table 9:** Normative data for the PSOC Efficacy Subscale in mothers with infants aged 0–9 months (percentiles, raw score, Z, and T).

Percentile	Direct Scoring	*Z*	*T*
5.0	26.05	−1.71	32.9
10.0	28.0	−1.38	36.2
15.0	30.0	−1.03	39.7
20.0	31.0	−0.86	41.4
25.0	32.0	−0.69	43.1
30.0	33.0	−0.52	44.8
35.0	34.0	−0.35	46.5
40.0	35.0	−0.18	48.2
45.0	36.0	−0.01	49.9
50.0	36.5	0.08	50.8
55.0	37.0	0.16	51.6
60.0	38.0	0.33	53.3
65.0	38.0	0.33	53.3
70.0	39.0	0.5	55.0
75.0	40.0	0.67	56.7
80.0	41.0	0.84	58.4
85.0	42.0	1.01	60.1
90.0	44.0	1.36	63.6
95.0	45.0	1.53	65.3

**Table 10:** Normative data for the PSOC Satisfaction Subscale in mothers with infants aged 0–9 months (percentiles, raw score, Z, and T).

Percentile	Raw Score	*Z*	*T*
5.0	32.0	−1.58	34.2
10.0	34.0	−1.24	37.6
15.0	36.0	−0.91	40.9
20.0	37.0	−0.74	42.6
25.0	38.0	−0.57	44.3
30.0	38.0	−0.57	44.3
35.0	40.0	−0.24	47.6
40.0	40.0	−0.24	47.6
45.0	41.0	−0.07	49.3
50.0	42.0	0.1	51.0
55.0	43.0	0.27	52.7
60.0	43.0	0.27	52.7
65.0	44.0	0.43	54.3
70.0	45.0	0.6	56.0
75.0	46.0	0.77	57.7
80.0	46.0	0.77	57.7
85.0	47.0	0.94	59.4
90.0	49.0	1.27	62.7
95.0	50.0	1.44	64.4

## Data Availability

The data presented in this study were obtained from the ‘Parental/Mother-Child Psychological Support Programme’©, and are available upon request to the corresponding author, subject to third-party permission.
